# Dietary Glutamate Supplementation Ameliorates Mycotoxin-Induced Abnormalities in the Intestinal Structure and Expression of Amino Acid Transporters in Young Pigs

**DOI:** 10.1371/journal.pone.0112357

**Published:** 2014-11-18

**Authors:** Jielin Duan, Jie Yin, Miaomiao Wu, Peng Liao, Dun Deng, Gang Liu, Qingqi Wen, Yongfei Wang, Wei Qiu, Yan Liu, Xingli Wu, Wenkai Ren, Bie Tan, Minghong Chen, Hao Xiao, Li Wu, Tiejun Li, Charles M. Nyachoti, Olayiwola Adeola, Yulong Yin

**Affiliations:** 1 Scientific Observing and Experimental Station of Animal Nutrition and Feed Science in South-Central, Ministry of Agriculture, Hunan Provincial Engineering Research Center of Healthy Livestock, Key Laboratory of Agro-ecological Processes in Subtropical Region, Institute of Subtropical Agriculture, Chinese Academy of Sciences, Changsha, Hunan 410125, China; 2 Department of Animal Nutrition, Fujian Aonong biotechnology corporation, Xiamen, Fujian 361007, China; 3 Research and Development Center, Twins Group Co., Ltd, Nanchang, Jiangxi 330096, China; 4 Hunan New Wellful Co., LTD, Changsha, Hunan, 410001, China; 5 Department of Animal science, University of Manitoba, Winnipeg, Man, R3T 2N2 Canada; 6 Department of Animal Science, Purdue University, West Lafayette, IN 47907, United States of America; 7 Southwest Collaborative Innovation center of swine for quality & safety, 211#211Huiming Road, Wenjiang district, Chengdu, China; German Institute of Human Nutrition Potsdam-Rehbrücke, Germany

## Abstract

The purpose of this study was to investigate the hypothesis that dietary supplementation with glutamic acid has beneficial effects on growth performance, antioxidant system, intestinal morphology, serum amino acid profile and the gene expression of intestinal amino acid transporters in growing swine fed mold-contaminated feed. Fifteen pigs (Landrace×Large White) with a mean body weight (BW) of 55 kg were randomly divided into control group (basal feed), mycotoxin group (contaminated feed) and glutamate group (2% glutamate+contaminated feed). Compared with control group, mold-contaminated feed decreased average daily gain (ADG) and increased feed conversion rate (FCR). Meanwhile, fed mold-contaminated feed impaired anti-oxidative system and intestinal morphology, as well as modified the serum amino acid profile in growing pigs. However, supplementation with glutamate exhibited potential positive effects on growth performance of pigs fed mold-contaminated feed, ameliorated the imbalance antioxidant system and abnormalities of intestinal structure caused by mycotoxins. In addition, dietary glutamate supplementation to some extent restored changed serum amino acid profile caused by mold-contaminated feed. In conclusion, glutamic acid may be act as a nutritional regulating factor to ameliorate the adverse effects induced by mycotoxins.

## Introduction

Mycotoxins are a group of structurally diverse secondary metabolites produced by a wide variety of fungal species and are commonly detected in cereal crops and cereal-based food products in temperate regions [1]–[3]. According to the numerous well-designed experiments, mycotoxins are absorbed into the metabolic cycle by a paracellular pathway through the tight junctions [4], and then exert acute and chronic physiology and immune toxicity on animals and humans [5], [6]. The ingestion of mycotoxins-contaminated feed lowers animal growth performance and meat quality [7], simultaneously alters gene expression [8], [9] and decreases the activity of intestinal glucose transporters [10]. Meanwhile, mycotoxins inhibit the proliferation of intestinal cells by inducing oxidative DNA damage and G1-phase arrest [11], cause severe inflammatory reaction [12],and unbalance the antioxidant system [13], [14] which play important roles in protecting our body against reactive oxygen species (ROS) [15]. Intestinal barrier dysfunction caused by mycotoxins [16] allows exogenous pathogenic antigens invasion, such as natural toxins and additional mycotoxins, which compromise intestinal homeostasis [17]. In contrast to normal feed or single-toxin contaminated feed, mold-contaminated feed contains multiple mycotoxins, such as aflatoxin B1 (AFB1), deoxynivalenol (DON), α-zearalenone (α-ZEA), ochratoxins (OCH), toxin-2 [18], [19]. Such multi-mycotoxin-contaminated feed plausibly imposes more serious damage on animals than the consumption of any single mycotoxin alone. Thus, contamination of feed by mixed myxotoxins greatly affects the health and economic stability of many farm industries, including swine production.

Glutamate is an important functional amino acid because of its physiological and immune contributions [20]. Glutamate serves as a pivotal substrate for other biological active molecules, including glutamine, glutathione, proline, and arginine [21], [22]. Considerable information from ongoing investigations in young pigs, preterm infants, and adult humans has shown that dietary glutamate is extensively metabolized in the intestine [23], [24]. Oxidation of glutamate in enterocytes is a major metabolic fate to product major oxidative fuels [25], [26] which are indispensable for intestinal cells proliferation and intestinal integrity and function [27]–[29]. In addition, glutamate locates at the center of disposal of amino acid and protein metabolism [30]. Therefore, glutamate plays important roles in facilitating protein biosynthesis and turnover, regulating gene expression, and enhancing immunity [22]. Many studies have demonstrated that dietary supplementation with glutamate restores mucous circulation and metabolism of amino acids as well as prevents the apoptosis of enterocytes [31], [32]. Akiba et al. have reported that L-glutamate enhances mucosal defenses by preventing cellular injury in small intestine [33]. Therefore, given the immune and physiological functions of glutamate, dietary supplementation with glutamate is rationally served as a promising approach to protect animals and humans from toxins exposure. Although many studies have reported some methods to reduce the presence of mysotoxin like physical and chemical degradation and the use of adsorbents, little information is known about glutamic acid to protect animals from the mycotoxins. Therefore, the current study was investigated to test the hypothesis that dietary supplementation with glutamate could mitigate the cytotoxic effects of mycotoxins on growing pigs.

## Materials and Methods

### Experimental design

Fifteen swine (Landrace×Large White) (ZhengHong Co., China) with a mean body weight (BW) of 55 kg were randomly divided into three treatment groups (n = 5/group): 1) the control group received basal feed; 2) the mycotoxin group received contaminated feed; 3) the glutamate group received contaminated feed and dietary supplementation with 2% glutamate (purity >99%, Beijing Chemclin Biotech, Beijing, China). Contaminated feed was mildewed clearly under ambient conditions (temperature 23–28°C, humidity 68–85%) as described by Liu et al [34] and the mycotoxins were detected by liquid chromatography (Beijing Taileqi, Beijing, China) (Table 1). The basal diets were formulated to meet or exceed the nutritional needs of growing pigs as recommended by the NRC (1998) (Table 2). The content of glutamate and other amino acids in basal diet was measured and presented in previous papers [35]–[37]. In the present study, control experiments were performed with diet containing no added amino acid. Indeed, we did not use alanine supplementation as a classical isonitrogenous control since the amount of glutamate used represents a negligible amount of supplemental nitrogen [38]. All pigs were allowed free access to water throughout the experimental period. This study approved by Laboratory Animal welfare Commission of the Institute of Subtropical Agriculture, Chinese Academy of Sciences [38].

**Table 1:** Mycotoxin content of contaminated and non-contaminated feed mixtures.

Catalogue	AFB1 (ppb)	ZEN (ppm)	OCH (ppb)	DON (ppm)	FB1 (ppm)	T-2 (ppm)
Limit of detection	0.05	0.01	0.5	0.1	0.05	0.1
Non-contaminatedfeed	undetected	0.821	3.6	1	0.6	undetected
Contaminated feed	0.62	0.573	11.39	3	2	undetected

Samples were collected at every preparing feed in each group and then mixed their together respectively. Among these mycotoxins, AFB1 means Aflatoxin B1 (ppb); ZEN means zearalenone (ppm); OCH means ochratoxin (ppb); DON means deoxynivalenol (ppm); FB1 means Fusarium B1 (ppm) and T-2 means T-2 fungal toxin (ppm).

**Table 2:** Composition and nutrient level of the basal diet.

Ingredients	Contents (%)	Nutrient Substance	Contents (%)
Corn	67.22	Crude protein	16
Soybean meal	21.8	Ca	0.6
Wheat bran	7.95	P	0.5
Limestone	1.03	CaHPO4	0.69
Lys	0.771	Salt	0.31
Met+Cys	0.584	Additive premix	1

Premix provided the following per kilogram of the diet: Sepiolite 8.072g; pig vitamin 750mg; FeSO4⋅H2O 317mg; CuSO4⋅5H2O 294mg; Antioxidants 200mg; MnSO4⋅H2O 172mg; ZnSO4⋅H2O 153mg; KI 24mg; Na2SeSO3 18mg.

### Sample collection

All blood samples were collected through a jugular vein from all of the pigs. Serum was separated by centrifugation at 1,500 g for 10 min at 4°C and stored at −20°C until analysis [39]. At day 60, the pigs were sacrificed and two gut samples were taken from both the mid-jejunum and mid-ileum. One of the gut sample (3 cm) was kept in 4% neutral buffered formalin for the determination of histomorphology and the other one (approximately 2 g) was immediately frozen in liquid nitrogen and stored at −70°C for subsequent analyses of gene expression [40].

#### Average daily weight gain (ADG) and feed conversion rate (FCR)

Pigs were weighed individually at day 0 and 60, and the feed consumption per pig was also recorded per pig at the same time to calculate the average daily weight gain (ADG; kg/pig/day) and the average feed conversion rate (RCR) [37].

#### Determination of serum T-SOD, GSH-Px and D-lactate activities, and DAO level

Serum Total superoxide dismutase (T-SOD) and glutathione peroxidase (GSH-Px) activities were measured using spectrophotometric kits in accordance with the manufacturer’s instructions (Nanjing Jiangcheng Biotechnology Institute, China) (PMID:21617969). Serum D-lactate was determined using an assay kit in accordance to the manufacturer’s instructions (Biovision Inc., USA). Diamine oxidase (DAO) level was measured according to Aarsen [41].

### Determination of serum amino acids

Twenty-seven amino acids were detected in serum based on our previous study [42]–[43] via LC-MS/MS (HPLC Ultimate3000 and 3200 Q TRAP LC-MS/MS): L-arginine, L-histidine, L-isoleucine, L-leucine, L-lysine, L-phenylalanine, L-methionine, L-threonine, L-tryptophan, L-valine, glycine, L-serine, L-tyrosine, L-asparagine, L-aspartic acid, L-citrulline, L-glutamic acid, L-glutamine, L-ornithine, L-cystine, L-homocystine, L-alanine, L-carnosine, hydroxy-L-proline, 1-methyl-L-histidine, 3-methyl-L-histidine, and L-proline.

#### Intestinal histomorphometry determination

The samples of the jejunum and ileum that had been kept in 4% neutral buffered formalin were processed using routine histological methods and mounted in paraffin blocks (PMID:22086211). Six-micrometer-thick sections were cut and stained with Masson’s trichrome. All specimens were examined under a light microscope (Nikon, Japan). Villus height and crypt depth were measured using an image-analysis system [44].

### RNA extraction and cDNA synthesis

Total RNA was isolated from liquid nitrogen-pulverized tissues as described above using TRIzol regent (Invitrogen, USA) and treated with DNase I (Invitrogen, USA) according to the manufacturer’s instructions [45]. The quality of RNA was checked by 1% agarose gel electrophoresis after staining with 10 µg/ml ethidium bromide. The RNA had an OD260:OD280 ratio between 1.8 and 2.0. First-strand cDNA was synthesized with oligo (dT) 20 and Superscript II reverse transcriptase (Invitrogen, USA).

### Quantification of mRNA by real-time PCR analysis

Primers were designed with Primer 5.0 based on the cDNA sequence of the pig to produce an amplification product (Table 3). GAPDH was used as a housekeeping gene to normalize target gene transcript levels. Real-time PCR was performed using SYBR Green PCR Mix, containing MgCl2, dNTP, and Hotstar Taq polymerase. Two µl of cDNA template was added to a total volume of 25 µl containing 12.5 µl SYBR Green mix and 1 µmol/l each of forward and reverse primers. We used the following protocol: (i) pre-denaturation (10 s at 95°C); (ii) amplification and quantification, repeated 40 cycles (5 s at 95°C, 20 s at 60°C); (iii) melting curve (60–99°C with a heating rate of 0.1°C S-1 and fluorescence measurement) (PMID:22086211).

**Table 3:** Primer pairs used in the RT-PCR reaction.

Gene	Accession No.	Nucleotide sequence of primers (5′–3′)
β-Actin	NM_001172909.1	F:CTGCGGCATCCACGAAACT
		R:AGGGCCGTGATCTCCTTCTG
SLC7A1	NM_001012613.1	F:TGCCCATACTTCCCGTCC
		R:GGTCCAGGTTACCGTCAG
SLC7A7	NM_001253680.1	F:TTTGTTATGCGGAACTGG
		R:AAAGGTGATGGCAATGAC
SLC1A1	NM_001164649.1	F:ATAGAAGTTGAAGACTGGGAAAT
		R:GTGTTGCTGAACTGGAGGAG
SLC5A1	NM_001164021.1	F:GGCTGGACGAAGTATGGTGT
		R:ACAACCACCCAAATCAGAGC

SLC7A1: solute carrier family 7 (cationic amino acid transporter, y+system), member 1; SLC7A7: solute carrier family 7 (amino acid transporter light chain, y+L system), member 7; SLC1A1: solute carrier family 1 (neuronal/epithelial high affinity glutamate transporter, system Xag), member 1; SLC5A1: solute carrier family 5 (sodium/glucose cotransporter), member 1. All these primer sequence was designed based on the sequence corresponding to the accession number described above.

### Statistical Analysis

All statistical analyses were performed using SPSS17.0 software. The normality and constant variance for date were tested by levene’s test, and then dates were subjected to one-way analysis of variance followed by the Duncan (D) multiple comparisons test. Values with different superscript letters are significantly different (P<0.05), while values with the same or no superscript letters are not significantly different (P>0.05). Data are expressed as the mean ± standard error of the mean (46).

## Result

### Growth performance

**Figure 1:**
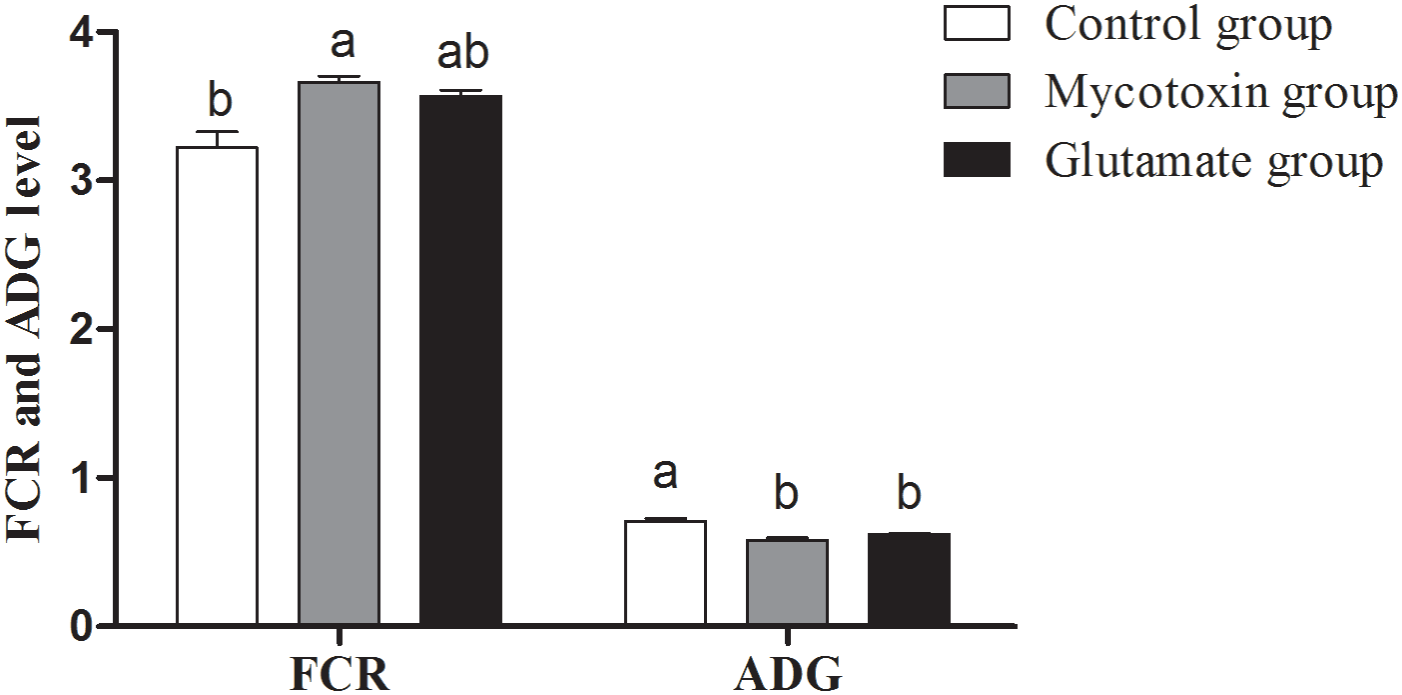
ADG and FCR in growing pigs fed mycotoxin-contaminated diets. The treatments consisted of a control group (n = 5) receiving uncontaminated feed, a mycotoxin group (n = 5) receiving mould-contaminated diet, and the glutamate group (n = 5) receiving mould-contaminated diet and 2% glutamate. The data with different letters in the same factor differ significantly (*P*<0.05), and same letters means no significant difference (*p*>0.05).

In current study, the ADG and FCR were measured to evaluate the growth performance and feed efficiency of young pig respectively. The ADG in the mycotoxin group between days 1 and 60 (0.58±0.02 kg/d) was lower (P<0.05) than those in the control group (0.71±0.02 kg/d) (Figure 1). Meanwhile, mold-contaminated feed (3.66±0.04) significantly increased (P<0.05) the FCR compared with control group (3.22±0.11). However, dietary supplementation with glutamate had little effects on the ADG (0.62±0.01 kg/d) and the FCR (3.57±0.04) compared with mycotoxin group. The ADG in glutamate group was significant lower (P<0.05) than those in the control group.

### Determination of serum T-SOD and GSH-Px activity

**Figure 2:**
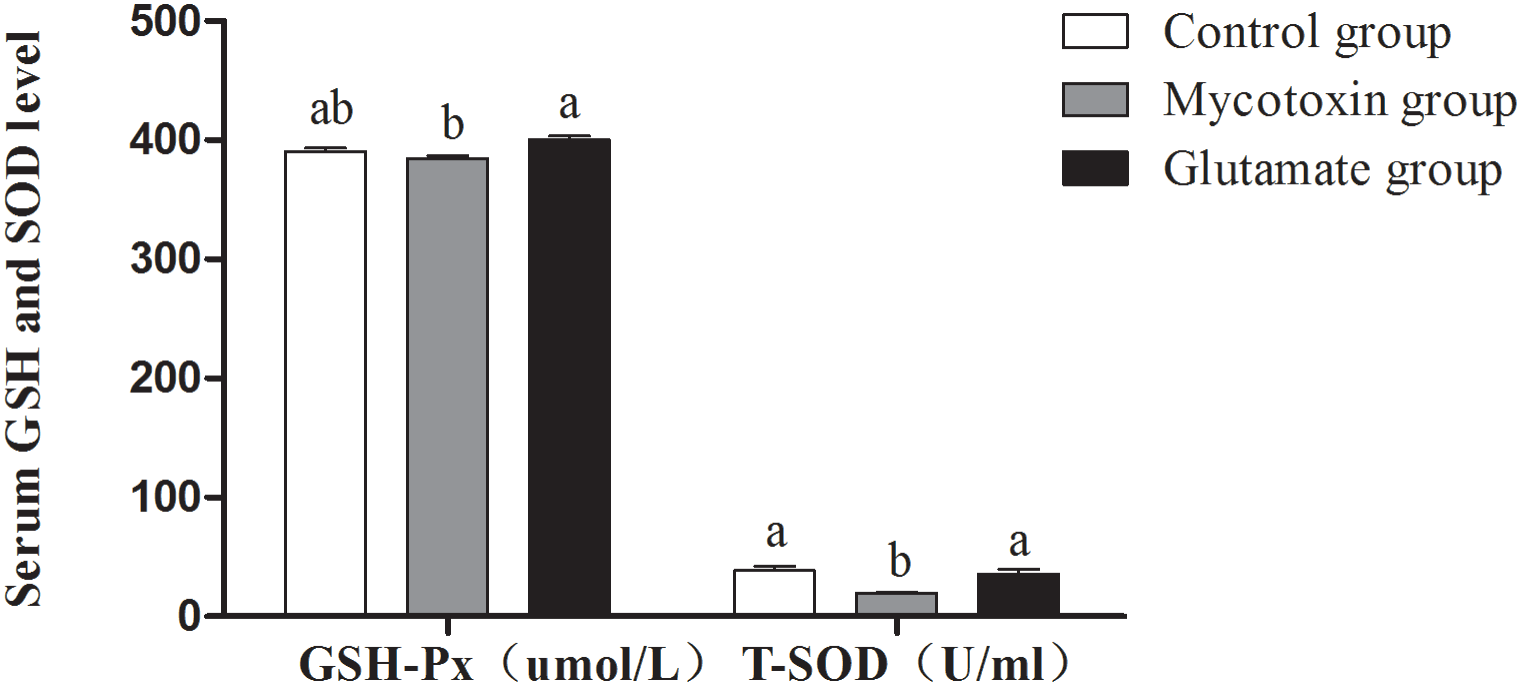
Serum GSH-Px and T-SOD activities in growing pigs fed mycotoxin-contaminated feed. The treatments consisted of a control group (n = 5) receiving uncontaminated feed, a mycotoxin group (n = 5) receiving mould-contaminated diet, and the glutamate group (n = 5) receiving mould-contaminated diet and 2% glutamate. The data with different letters in the same factor differ significantly (*P*<0.05), and same letters means no significant difference (*p*>0.05).

SOD and GSH-Px are two major antioxidant enzymes to scavenge the excessive internal reactive oxygen species (ROS) which exert radical-mediates damages to biological macromolecules (proteins, lipids and DNA) [42], therefore the activities of SOD and GSH-Px are plausibly regarded as a mark reflecting redox of organism [15]. In this study, compared with the control group, mold-contaminated feed significantly decreased T-SOD activity (P<0.05) and GSH-Px activity (P>0.05) (Figure 2). However, dietary supplementation with glutamic acid significantly augmented (P<0.05) serum T-SOD and GSH-Px activities compared with the mycotoxin group, and the serum T-SOD and GSH-Px activities in glutamate group were restored to parallel with the control group (Figure 2).

### Determination of serum D-lactate and DAO

**Figure 3:**
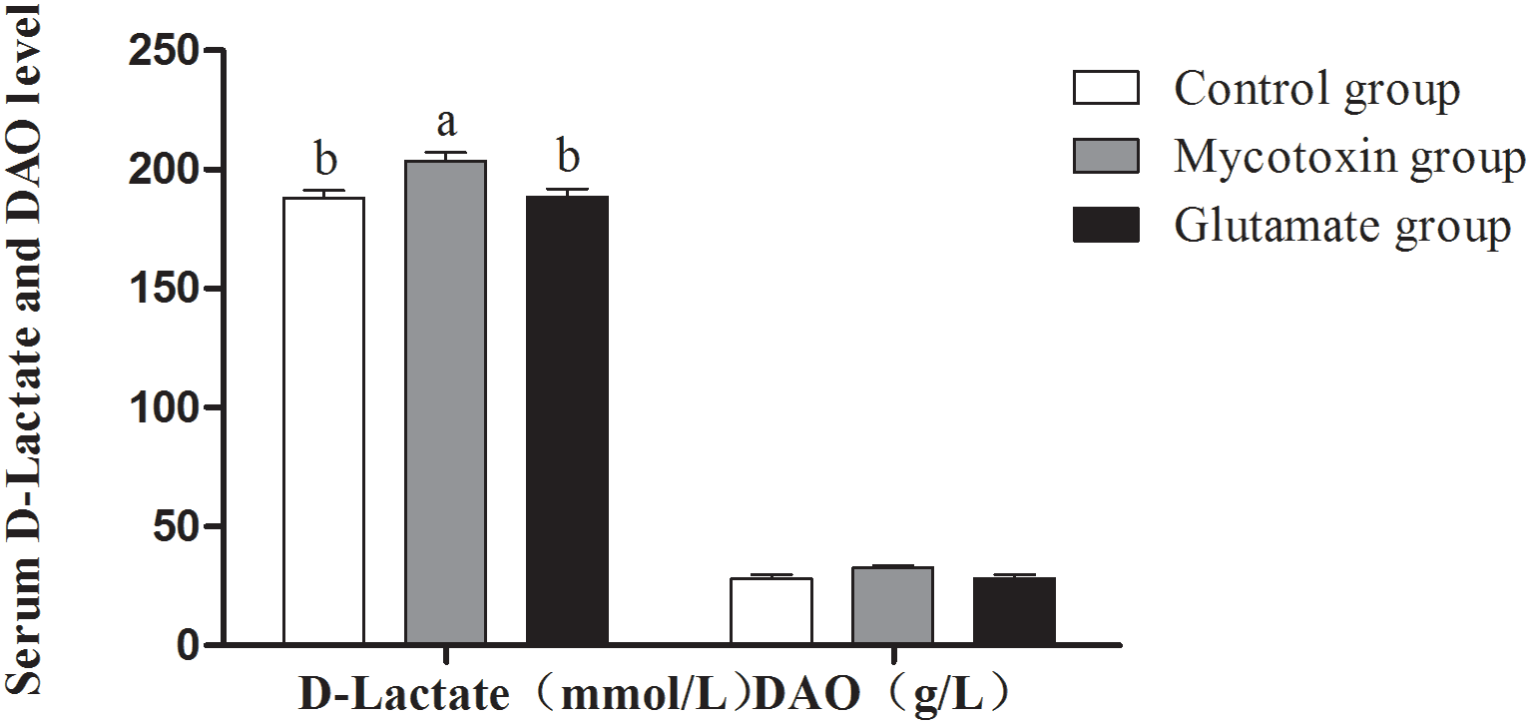
Serum D-Lactate and DAO activities of growing pigs fed mycotoxin-contaminated feed. The treatments consisted of a control group (n = 5) receiving uncontaminated feed, a mycotoxin group (n = 5) receiving mould-contaminated diet, and the glutamate group (n = 5) receiving mould-contaminated diet and 2% glutamate. The data with different letters in the same factor differ significantly (*P*<0.05), and same letters means no significant difference (*p*>0.05).

In this study, serum D-lactate levels and DAO activity were measured to evaluate intestinal integrity. As shown in figure 3, after pigs exposed to contaminated feed, the D-lactate levels were significantly increased (P<0.05). However, the D-lactate levels in the glutamate group were significantly lowered (P<0.05) than those in the mycotoxin group. There was no significant difference in the level of DAO among all groups (Figure 3).

### Microscopic Evaluation

**Figure 4:**
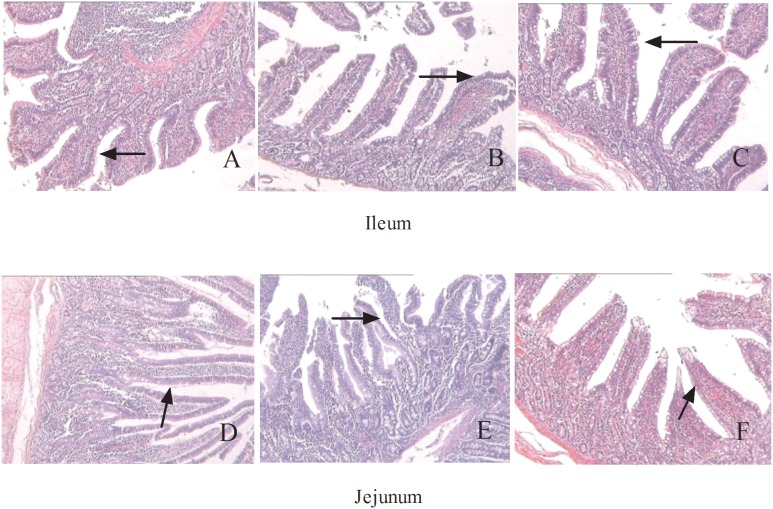
Histological evaluation of intestinal tissues (HE×100) in growing pigs fed mould-contaminated feed. The treatments consisted of a control group (n = 5) receiving uncontaminated feed, a mycotoxin group (n = 5) receiving mould-contaminated diet, and the glutamate group (n = 5) receiving mould-contaminated diet and 2% glutamate. Fig. 4A and D represented control group and Fig. 4B and D represented contaminated group and Fig. 4C and F represented glutamate group. There is no histological damage observed in the control group (Fig. 4A and D). In mycotoxin group, the villus was scattered and desquamated seriously in jejunum and ileum (Fig. 4B and D). A greater villus in jejunum and ileum was observed in glutamate group (Fig. 4C and F).

In this study, compared with control group, pigs with contaminated feed remarkably increased (P<0.05) in the villus height and crypt depth in the ileum and jejunum, while there was no difference in the ratio of villus height to crypt depth in the ileum and jejunum (Table 4). However, glutamate supplementation significantly decreased (P<0.05) ileum the crypt depth and jejunum villus height, but had no significant difference on the ratio of villus height to crypt depth in the ileum and jejunum (Table 4). As shown in Figure 4, no histological damage was observed in the ileum (Fig. 4A) and jejunum (Fig. 4D) in the control group. In the contaminated feed group, villi in the ileum (Fig. 4B) and jejunum (Fig. 4E) were scattered and desquamated. The glutamate group showed larger villi in both the ileum (Fig. 4C) and jejunum (Fig. 4F).

**Table 4:** Effect of dietary supplementation with glutamate on morphological characteristics in intestinal tissues in pigs fed experimental diets.

Catalogue	Control group	Mycotoxin group	Glutamate group
Ileum villus height (µm)	255.45±4.89b	356.15±8.08a	332.03±11.96a
Ileum crypt depth (µm)	120.00±1.56b	216.97±7.17a	139.90±8.75b
Jejunum villus height (µm)	295.08±6.39b	343.30±16.04a	298.65±7.05b
Jejunum crypt depth (µm)	99.13±3.90b	132.45±6.84a	113.35±6.31ab
Ileum V/C	2.97±0.18	2.48±0.20	2.68±0.22
Jejunum V/C	2.41±0.39	2.2±0.41	2.22±0.65
Ileum goblet cells number	32.00±1.92a	23.25±3.38b	25.00±2.47ab
Jejunum goblet cells number	16.50±1.50a	12.75±0.86b	16.33±0.62a
Ileum lymphocyte number	223.75±17.67	179.75±26.71	214.00±26.05
Jejunum lymphocyte number	286.00±27.25	248.20±18.83	226.80±11.38

The treatments consisted of a control group (n = 5) receiving uncontaminated feed, a mycotoxin group (n = 5) receiving mould-contaminated diet, and the glutamate group (n = 5) receiving mould-contaminated diet and 2% glutamate. Villus height and crypt depth were measured using an image-analysis system. Among these indexes, Ileum C/V means the ration of ileal villus height to crypt depth and Jejunum C/V means the ration of jejunal villus height to crypt depth. The data with different letters in the same row differ significantly (P<0.05), and same letters mean no significant difference (p>0.05).

### Serum amino acid parameters

As shown in Table 5, the consumption of contaminated feed resulted in decreases in the serum levels of some amino acids. Compared with control group, contaminated feed significantly decreased (P<0.05) the serum concentrations of L-glutamine, L-proline, 1-methyl-L-histidine, hydroxy-L-proline and L-tyrosine and simultaneously the concentrations of L-glutamic acid and amino acids related to its metabolism (L-ornithine) in mycotoxin group tended to be lower (P>0.05), while the level of L-citrulline was significantly higher than that in the control group (P<0.05). However, dietary supplementation with glutamate significantly restored (P<0.05) the serum levels of 1-methyl-L-histidine, hydroxy-L-proline, L-homocystine, and L-histidine, and the levels of L-homocystine and L-histidine were even higher than those in the control group. In addition, the concentrations of L-glutamic acid and L-proline in the glutamate group tended to be higher (P>0.05) than those in the mycotoxin group.

**Table 5:** Effect of dietary supplementation with glutamate on concentration of serum amino acid parameters in growing pigs fed mycotoxin-contaminated feed.

Item	The molecularformula	Control group(ug/ml)	Mycotoxin group (ug/ml)	Glutamate group (ug/ml)
L-arginine	C6H14N4O2	23.34±0.32	20.79±2.82	20.43±0.32
L-histidine	C11H17N3O4	32.69±3.53b	32.76±2.70b	45.42±5.61a
L-isoleucine	C6H13NO2	10.85±0.98	10.74±0.79	9.91±0.26
L-leucine	C6H13NO2	22.48±0.21	22.61±0.89	22.05±0.64
L-lysine	C6H14N2O2	16.69±1.41	16.03±0.80	14.41±1.56
L-phenylalanine	C5H11O2NS	12.16±0.87	11.03±1.52	10.63±0.24
L-methionine	C9H11NO2	32.93±0.90b	31.23±5.41b	50.28±3.50a
L-threonine	C4H9NO3	10.17±0.34	10.08±0.94	9.57±0.94
L-tryptophan	C11H12N2O2	6.87±0.36	6.31±0.45	6.15±0.89
L-valine	C5H11NO2	20.30±1.81	22.17±1.73	22.39±2.61
Glycine	C2H5NO2	87.83±3.25a	67.02±3.11b	74.33±2.37ab
L-serine	C3H7NO3	10.05±1.73	10.19±0.55	10.62±0.96
L-tyrosine	C2H7NSO3	23.25±1.24a	17.60±0.86b	17.13±2.08b
L-asparagine	C9H11NO3	2.84±0.28	3.26±0.40	3.31±0.09
L-aspartic acid	C4H8N2O3	1.72±0.28b	1.67±0.09b	2.76±0.48a
L-citrulline	C4H7NO4	10.13±0.91b	17.70±1.09a	13.15±0.83b
L-glutamic acid	C6H13N3O3	57.25±3.09	49.31±2.85	53.12±8.78
L-glutamine	C5H9NO4	7.20±0.67a	3.90±0.30b	4.90±0.38b
L-ornithine	C5H10N2O3	13.09±0.92	11.02±1.21	12.05±2.14
L-cystine	C5H12N2O2	0.49±0.12	0.32±0.06	0.43±0.10
L-homocystine	C3H7NO2S	0.18±0.01b	0.15±0.02b	0.25±0.02a
L-alanine	C4H9NO2S	44.03±6.70	44.66±3.67	48.85±9.85
L-carnosine	C3H7NO2	0.76±0.18	0.69±0.09	0.61±0.25
Hydroxy-L-proline	C5H9NO3	80.85±5.76a	29.31±4.09c	53.18±4.70b
1-methyl-L-histidine	C7H11N3O2	0.70±0.05a	0.49±0.03b	0.69±0.06a
3-methyl-L-histidine	C7H11N3O2	1.64±0.10ab	1.46±0.08b	1.74±0.12a
L-proline	C5H9NO2	46.13±0.33a	20.37±0.36b	25.95±2.68b

The treatments consisted of a control group (n = 5) receiving uncontaminated feed, a mycotoxin group (n = 5) receiving mould-contaminated diet, and the glutamate group (n = 5) receiving mould-contaminated diet and 2% glutamate. The data with different letters in the same row differ significantly (P<0.05), and same letters mean no significant difference (p>0.05).

### Amino acid transporter gene expression

**Figure 5:**
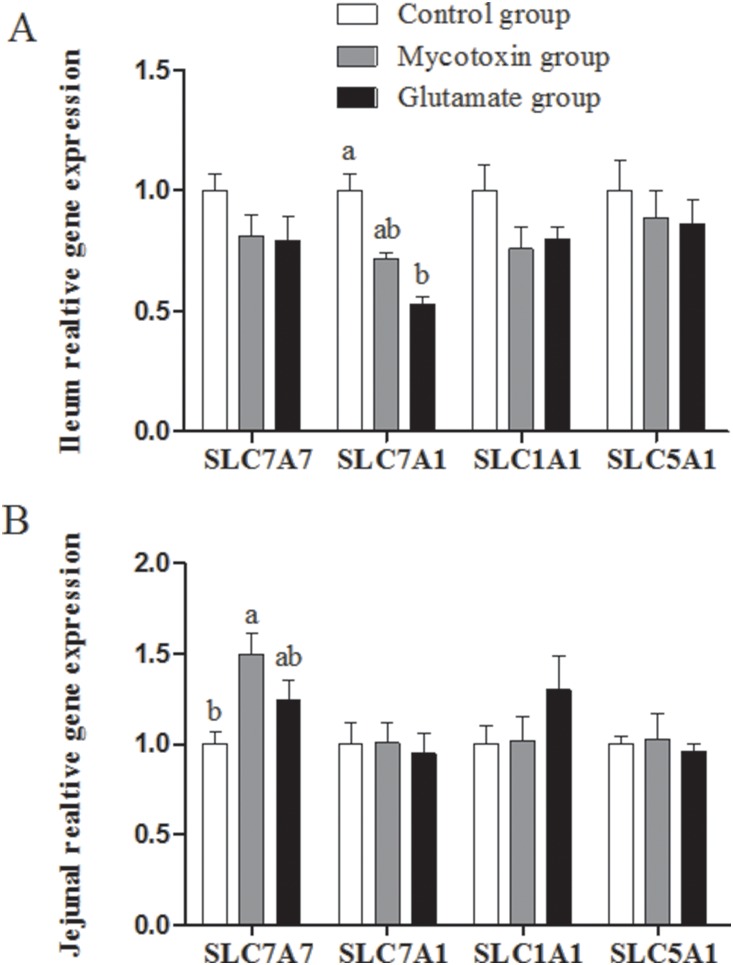
Effect of dietary supplementation with glutamate on elative mRNA abudances in ileum (A) and jejunum (A) of growing pigs fed mould-contaminated feed. The treatments consisted of a control group (n = 5) receiving uncontaminated feed, a mycotoxin group (n = 5) receiving mould-contaminated diet, and the glutamate group (n = 5) receiving mould-contaminated diet and 2% glutamate. SLC7A1: solute carrier family 7 (cationic amino acid transporter, y+system), member 1; SLC7A7: solute carrier family 7 (amino acid transporter light chain, y+L system), member 7; SLC1A1: solute carrier family 1 (neuronal/epithelial high affinity glutamate transporter, system Xag), member 1; SLC5A1: solute carrier family 5 (sodium/glucose cotransporter), member 1. The data with different letters in the same row differ significantly (*P*<0.05), and same letters means no significant difference (*p*>0.05).

The ileal solute carrier family 7 (amino acid transporter light chain, y+L system), member 7 (SLC7A7), solute carrier family 7 (cationic amino acid transporter, y+system), member 1 (SLC7A1), solute carrier family 1 (neuronal/epithelial high affinity glutamate transporter, system Xag), member 1 (SLC1A1), and solute carrier family 5 (sodium/glucose co-transporter), member 1 (SLC5A1) mRNA abundances in the mycotoxin group exhibited no significant difference compared to those in the control group (Figure 5A). Contaminated feed also exhibited no effects on mRNA abundances of these genes in the jejunum excepting significantly increased (P<0.05) mRNA abundance of SLC7A7 (Figure 5B). There was no difference about the mRNA expression of these transporters between mycotoxin group and glutamate group in the jejunum and ileum (P>0.05).

## Discussion

Glutamate is an important versatile functional amino acid because of its nutritional and immune contributions. Many well-designed studies have shown that glutamate performs critical roles in regulating intestinal health and maintaining intestinal homeostasis by providing pivotal oxidative fuels that are indispensable for enterocytes proliferation and turnover and enhancing intestinal barrier function [21], [32]. Although dietary glutamate is crucial for intestinal health, little is known about its role in protecting the gut from toxin-induced injury. Thus, we conducted the experiment to explore whether dietary supplementation with glutamate could attenuate the cytotoxic effects of mycotoxins on growing pigs.

In current study, liquid chromatography determination showed that AFB1 (0.62 ppb), OCH (11.39 ppb), DON (3 ppm), and FB1 (2 ppm) were the main mycotoxins in moldy feed. Although the AFB1 and OCH levels were quite low (the concentrations were still higher than those in the control group), there is as yet no method suitable for determining whether their co-effects might produce more serious impairments than either compound alone. A growing number of studies have shown that consumption of mycotoxins decreases ADG, feed intake, and thus lower animal performance [46]. Indeed, in our study, contaminated feed lowered pig growth performance and feed efficiency with significantly decreasing ADG and increasing FCR respectively, which parallelized with previous report that DON significantly decreases pig growth performance [45]. According to previous studies, ingestion of mycotoxins can remarkably damage intestinal structure, gut barrier and intestinal immunity, leading to the compromise intestinal function [4], [12,]. Thus adverse effects of mycotoxins on intestinal function maybe account for the pig growth-suppression caused by contaminated feed in our study. However, supplementation with 2% glutamate failed to mitigate the growth-suppression caused by mycotoxins. Similarly, previous reports have shown that dietary supplementation with arginine and glutamine also fails to alleviate the growth-suppression induced by mycotoxins in growing pig. Thus, we speculate the reason may be that the supplemental glutamate concentration in current study is insufficient to overwhelm the mycotoxin-induced growth-suppression. As a result, to elucidate this point, further study need to be carried out.

Poor growth performance is relevant to injuries of intestinal absorption and intestinal function caused by mycotoxins. Many of studies have demonstrated that mycotoxins can damage intestinal structure, impair intestinal carrier function and unbalance antioxidant system [13], [45], [46]. Similar to the previous studies, fed contaminated feed impaired the intestinal structure, antioxidant system and intestinal barrier function in our experiment. Intestinal histological and morphological impairment and intestinal barrier dysfunction lead to poor nutrient absorption, and then lower animal performance and in this study growth-suppression induced by mycotoxins have indirectly demonstrated this point. Intriguingly, according to previous investigations, supplementation with amino acid and peptide preparations may counteract the toxic effects of mycotoxins in mice and pigs [47]. For example, supplementation with protein and amino acids overcomes the mycotoxicoses [47], [48] and the addition of proline exhibits a beneficial effect on the jejunum impairment induced by DON [49]. As expected, dietary supplementation with glutamate not only remarkably improved structure of the intestine (based on histological and morphological findings), but also restored intestinal barrier function and antioxidant system with decreased serum D-lactate level and increased serum SOD and GSH-Px levels which can scavenge excessive ROS. These results have demonstrated certain beneficial roles of glutamate restore impaired intestinal function in pigs after challenge with contaminated feed. In mammals, glutamate plays an important role in the synthesis and turnover of non-essential amino acids and protein in the gut [50] and also provides major oxidative fuels, which play critical roles in reducing experimental intestinal hyper-permeability and facilitating gut integrity and function [51]. Meanwhile, glutamate is a precursor for glutathione and N-acetylglutamate in enterocytes and glutathione is involved in intestinal redox state and in the detoxication process and simultaneously performs pivotal roles in regulating intestinal function [52]. Thus, considering the versatile beneficial function of glutamate in intestine, it is plausible that dietary supplementation with glutamate may to some extent protect the intestinal homeostasis from contaminated feed.

Serum amino acids are the substrate for nutritional anabolism and catabolism, playing important roles in immune response and growth performance. In current study, the contaminated feed decreased some of the serum amino acid concentration. In particular, the serum level of L-glutamine, L-proline, 1-methyl-L-histidine, hydroxy-L-proline, and L-tyrosine were significantly decreased and L-glutamic acid and amino acids related to its metabolism (L-ornithine) tended to be lower. Meloche et al. have reported that T 2 toxin reduces amino acid uptake as well as the plasma amino acid concentration [53], which is consistent with our present results. However, dietary supplementation with glutamate restores L-histidine, L-methionine, L-homolysine, 1-methyl-L-histidine and 3-methyl-L-histidine levels, but fails to restore serum L-glutamate, L-glutamine and L-citrulline levels. Similarly, Boutry et al have also reported glutamate supplementation fail to reverse decreased glutamate, glutamine and citrulline concentrations in plasma in endotoxemia. A possible explanation for these results is that both glutamate and other amino acids (glutamine, ornithine and proline) are accumulated in the intestinal mucosa and then preferentially are used for oxidative metabolism to produce ATP and protein biosynthesis in enterocytes [21] to repair injured intestinal function induced by mycotoxins, rather than are transferred to the bloodstream. The levels of serum amino acids are related to amino acid transporters because of amino acid-sensor and -carrier function of amino acid transporters [54]. Before used for metabolism, luminal amino acids must be transported into the bloodstream through amino acid transporters (e.g., SLC7A7, SLC7A1, SLC1A1, and SLC5A1) which are extensively located at the intestinal mucosa. In current study, contaminated feed exhibited no significant effects on the mRNA expression of intestinal amino acid transporters. However, previous studies have indicated that mycotoxins inhibit amino acid transporters expression [55]. The discrepancy with other studies may be growing pigs that highly adapt to contaminated feed. However, dietary supplementation with glutamate exhibits no benefits to amino acid transporters expression after contaminated feed challenge. Previous study have reported that dietary supplementation with arginine or N-carbamylglutamate up-regulates of SLC1A1 gene expression or vascular endothelial growth factor or and mTOR Signaling Activity [56]–[59]. However, our previous investigation has indicated that supplementation with arginine also fails to up-regulate intestinal amino acid transporters [37]. Thus, with respected to this contradictory results, we speculate that the reason may be due to animal model, duration of feeding and the concentration of glutamate supplementation. However, further studies should be carried out to elucidate this point in detail.

In conclusion, treatment of pigs with mold-contaminated feed has adverse effects on growth performance, structure of the intestine (histology, morphology and barrier function), and serum amino acid profile. Glutamate likes other functional amino acids can improve animal health [60]–[77]. Dietary supplementation with glutamate partially counteracts the impairments induced by mycotoxins. Therefore, glutamate may be useful as a nutritional regulating factor to alleviate the adverse effects of mycotoxins.
